# A genome-wide cross-trait analysis identifies shared loci and causal relationships of obesity and lipidemic traits with psoriasis

**DOI:** 10.3389/fimmu.2024.1328297

**Published:** 2024-03-14

**Authors:** Yuan Wu, Mengfen Huang, Xueru Chen, Jingjing Wu, Li Li, Jianan Wei, Chuanjian Lu, Ling Han, Yue Lu

**Affiliations:** ^1^ The Second Clinical Medical College, Guangzhou University of Chinese Medicine, Guangzhou, China; ^2^ The First Clinical Medical College, Guangzhou University of Chinese Medicine, Guangzhou, China; ^3^ Guangdong-Hong Kong-Macau Joint Lab on Chinese Medicine and Immune Disease Research, The Second Affiliated Hospital of Guangzhou University of Chinese Medicine (Guangdong Provincial Hospital of Chinese Medicine), Guangzhou, China; ^4^ Guangdong Provincial Key Laboratory of Clinical Research on Traditional Chinese Medicine Syndrome, The Second Affiliated Hospital of Guangzhou University of Chinese Medicine, Guangzhou, China; ^5^ State Key Laboratory of Dampness Syndrome of Chinese Medicine, The Second Affiliated Hospital of Guangzhou University of Chinese Medicine, Guangzhou, China

**Keywords:** psoriasis, obesity, lipidemic, genome-wide cross-trait analysis, Mendelian randomization

## Abstract

**Background:**

Obesity and dyslipidemia, major global health concerns, have been linked to psoriasis, but previous studies faced methodological limitations and their shared genetic basis remains unclear. This study examines various obesity-related and lipidemic traits as potential contributors to psoriasis development, aiming to clarify their genetic associations and potential causal links.

**Methods:**

Summary statistics from genome-wide association studies (GWAS) conducted for obesity-related traits (body mass index (BMI), waist-to-hip ratio (WHR), and waist-to-hip ratio adjusted for the body mass index (WHR_adj_BMI)) and lipidemic traits (high-density lipoprotein (HDL), LDL, triglyceride (TG), total Cholesterol (TC), apolipoprotein A1 (apoA1), apolipoprotein B (apoB), and apolipoprotein E (apoE)) and psoriasis, all in populations of European ancestry, were used. We quantified genetic correlations, identified shared loci and explored causal relationship across traits.

**Results:**

We found positive genetic correlation between BMI and psoriasis (r_g_=0.22, p=2.44×10^-18^), and between WHR and psoriasis (r_g_=0.19, p=1.41×10^-12^). We further found the positive genetic correlation between psoriasis and WHR_adj_BMI(r_g_=0.07, p=1.81×10^-2^) the genetic correlation, in while the effect of BMI was controlled for. We identified 14 shared loci underlying psoriasis and obesity-related traits and 43 shared loci between psoriasis and lipidemic traits via cross-trait meta-analysis. Mendelian randomization (MR) supported the causal roles of BMI (IVW OR=1.483, 95%CI=1.333-1.649), WHR (IVW OR=1.393, 95%CI=1.207-1.608) and WHR_adj_BMI (IVW OR=1.18, 95%CI=1.047-1.329) in psoriasis, but not observe any significant association between lipidemic traits and the risk of psoriasis. Genetic predisposition to psoriasis did not appear to affect the risk of obesity and lipidemic traits.

**Conclusions:**

An intrinsic link between obesity-related traits and psoriasis has been demonstrated. The genetic correlation and causal role of obesity-related traits in psoriasis highlight the significance of weight management in both the prevention and treatment of this condition.

## Introduction

Psoriasis is an immune-mediated, chronic inflammatory skin disease, characterized by scaly, hardened and erythematous patches on the skin. It affects approximately 2% to 3% of the global population (1, 2). Severe cases of psoriasis can result in significant impairments in both physical and mental health, ultimately diminishing an individual’s overall quality of life (2–4). An increasing trend in psoriasis prevalence has been consistently reported in recent years (4, 5). Furthermore, psoriasis frequently coexists with other conditions, including metabolic syndrome (MetS), psychological disorders, cardiovascular disease (CAD), dyslipidemia and so on, which have also been implicated as potential risk factors for the development of psoriasis (6–9). Despite ongoing research efforts, the precise etiology and pathogenesis of psoriasis have not been fully elucidated. Therefore, it holds substantial clinical significance to identify potential molecular mechanism that could aid in the prevention, diagnosis, and treatment of psoriasis.

Obesity and dyslipidemia have emerged as major global health challenges due to their escalating prevalence and their association with numerous comorbidities, including CAD, diabetes, and psoriasis, leading to a substantial burden on individuals and society (10–13). Observational studies and meta-analyses in the existing literature have consistently demonstrated a positive correlation between obesity and the incidence of psoriasis (14, 15). In a population-based twin study, a robust correlation between psoriasis and both body mass index (BMI) and obesity was observed, speculating that a portion of the association between psoriasis and obesity could be attributed to a shared genetic predisposition (16). Furthermore, clinical studies and meta-analyses have shown a significant elevation in the levels of very low-density lipoprotein (VLDL) and low-density lipoprotein (LDL) in patients with psoriasis (17, 18). Prior research has underscored the importance of studying the obese and lipid phenotype concerning psoriasis management and prognosis. However, there are methodological limitations of previous studies, including the potential for reverse causality and the presence of confounding factors.

Recent advances in large-scale genetic studies, coupled with advanced statistical approaches, enable us to overcome these limitations and uncover both shared and distinct genetic architectures, thereby enhancing our understanding of disease biology (19, 20). Several analytical components are integral to such an investigation: genetic correlation analysis for estimating genetic associations, cross-trait meta-analysis for identifying shared genetic loci, and Mendelian randomization (MR) to establish causal relationships. Although several MR studies have demonstrated the causal relationship between obesity, lipids, and psoriasis, comprehensive sensitivity analyses were not consistently performed to validate model assumptions (21, 22). Moreover, to the best of our knowledge, few prior attempts have been conducted to determine the genetic correlation and whether the genetic correlation can be attributed to specific loci or extends across the entire genome.

**Figure 1:**
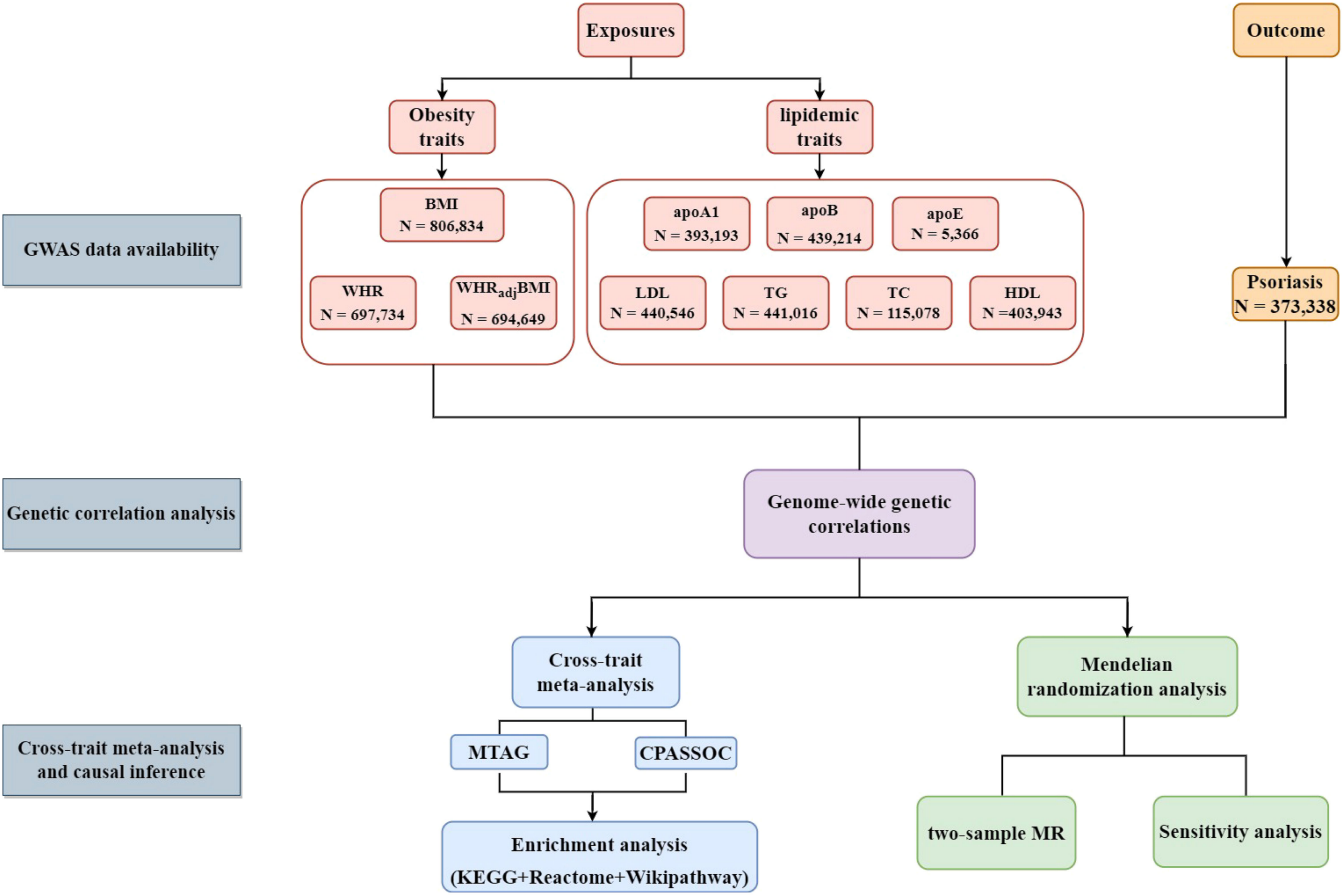
Flow-chart on the overall study design. (BMI, body mass index; WHR; waist-to-hip ratio; WHR_adj_BMI, waist-to-hip ratio adjusted for body mass index; apoA1, apolipoprotein A1; apoB, apolipoprotein B; apoE, apolipoprotein E; HDL, high-density lipoprotein; LDL, low-density lipoprotein; TC, total cholesterol; TG, triglycerides; CPASSOC, cross-phenotype association analysis; MTAG, Multi-trait analysis of GWAS; KEGG, Kyoto Encyclopedia of Genes and Genomes.).

Therefore, the present study aims to explore the shared architecture and the causal relationship between obesity-related traits, lipidemic traits, and psoriasis through comprehensive genetic analysis. In this study, obesity-related traits (BMI, waist-to-hip ratio (WHR), and waist-to-hip ratio adjusted for the body mass index (WHR_adj_BMI)) and lipidemic traits (high-density lipoprotein (HDL), LDL, triglyceride (TG), total cholesterol (TC), apolipoprotein A1 (apoA1), apolipoprotein B (apoB), and apolipoprotein E (apoE)) were examined as exposures in the development of psoriasis. The conceptual framework is illustrated in **Figure 1**.

## Methods

We conducted the current study by utilizing large-scale genome-wide association studies (GWAS) summary statistics and novel statistical genetic approaches. We incorporated genetic data related to obesity and lipidemic traits to ensure optimal alignment with psoriasis. In order to mitigate potential bias arising from population stratification, all genetic data were limited to the European population.

### GWASs data source

For BMI, the largest GWAS to date was conducted by meta-analyzing data from UK Biobank and GIANT consortium, which collectively included 806,834 individuals of European ancestry, 697,734 for WHR, and 694,649 for WHR_adj_BMI (23, 24). In each participating study, Single Nucleotide Polymorphisms (SNPs) were imputed using the Haplotype Reference Consortium (HRC) reference panel and subjected to rigorous filtering criteria, including an imputation quality score > 0.30, a call rate > 0.95, a minor allele frequency > 0.0001 and a P value for Hardy-Weinberg equilibrium > 10^−6^. After implementing quality control measures, genome-wide association testing was performed using a linear mixed model that was adjusted for variables including age, recruitment center, genotyping batches, and principal components. A fixed-effect inverse-variance-weighted meta-analysis was conducted to combine effect sizes across studies. To pinpoint independent top-associated SNPs, a PLINK clumping algorithm with a significance threshold of P< 1×10^−9^ and an LD window of ±5 Mb (r^2^>0.05) were first employed to form LD-based clumps, followed by a proximal conditional and joint testing to detect primary and secondary signals within each of the clumping-based loci (25).

The most extensive GWAS for lipids and apolipoproteins, encompassing HDL, LDL, TG, TC, apoA1, and apoB, was sourced from the UK Biobank (26), and apoE was obtained from a large-scale proteogenomic study (27). We acquired genetic associations for each trait in unrelated individuals of European ancestry (n=5,366-441,016). Analyses were adjusted for age and sex, and inverse rank normalization was used to standardize each trait. The mean set to 0, with a standard deviation (SD) of 1.

A total of 9,267 psoriasis cases were acquired from the FINNGEN Consortium and compared against 364,071 controls of European ancestry (28). All analyses utilized the human reference genome builds 37 (or hg19).

### Statistical analysis

#### Genetic correlation analysis

Genome-wide genetic correlations (r_g_) quantify the average sharing of genetic effect between two traits, independent of environmental confounders. The estimate ranges from -1 to 1, with -1 indicating a perfect negative genetic correlation and 1 indicating a perfect positive genetic correlation. It can be quantified using GWAS summary data through linkage-disequilibrium score regression (LSDC) (29, 30). We employed pre-computed LD scores derived from ~1.2 million common SNPs in European ancestry represented in the HapMap3 reference panel, commonly recognized as of high imputation quality.

#### Cross-trait meta-analysis

Cross-phenotype association analysis (CPASSOC) combines GWAS summary statistics from multiple related traits to identify variants associated with multiple traits across studies, while controlling population structure and cryptic relatedness (31). CAPSSOC offers two test statistics, S_Hom_ and S_Het_. S_Hom_ employs the fixed effect meta-analysis method and is most effective when genetic effect sizes are homogenous, which may not hold true especially when analyzing multiple traits. S_Het_ is an extension of S_Hom_ with improved power, accommodating heterogeneous effects of a trait from different study designs, environmental factors, or populations, as well as heterogeneous effects for different phenotypes, which is more commonly observed in practice. We applied pairwise S_Het_ to integrate summary statistics for each of the obesity and lipidemic traits with psoriasis. We applied PLINK clumping function parameters: (–clump-p 0.99 –clump-r^2^ 0.2 –clump-kb 500) to identify independent SNPs (25). Significant SNPs were defined as variants with P_single trait_<1×10^-3^ (for each single trait) and P_CPASSOC_<5×10^-8^ (for cross-traits). Multi-trait analysis of GWAS (MTAG) employs generalized inverse-variance-weighted meta-analysis for multiple associated traits and designed to detect novel genetic correlations for each trait (32). In brief, MTAG takes summary statistics from each GWAS as inputs and generates trait-specific effects for one common set of SNPs. P_mtag_<5×10^−8^ is considered as genome-wide significance level for MTAG (20). We performed an analysis using MTAG v.1.0.8 as a complementary method for cross-trait meta-analysis. For detailed functional annotation of the variants identified by cross-trait meta-analysis, we employed the Ensemble Variant Effect Predictor (VEP), together with eQTL dataset (https://www.eqtlgen.org/).

#### Exclusion of major histocompatibility complex region

Since the MHC region, defined as base positions 24,000,000 to 35,000,000 on chromosome 6 (GRCh37), was in a complex LD structure, which renders it susceptible to horizontal pleiotropy. As a result, we repeated the analysis after excluding SNPs from MHC region.

#### Enrichment analysis

To explore the biological pathways associated with the significant genes identified through cross-trait meta-analysis, we evaluated the enrichment of gene sets in the Kyoto Encyclopedia of Genes and Genomes (KEGG), Reactome database and Wikipathway by using the WebGestalt tool (33).

#### Mendelian randomization analysis

To investigate the causal relationship between each of the obesity and lipidemic traits and psoriasis, a two-sample MR was performed. For instrumental variables (IVs) selection, since properties of the MHC region violate the assumptions of MR, we excluded SNPs in MHC region for further MR analysis. Our primary MR analysis employed the inverse-variance weighted (IVW) approach, and we supplemented it with MR-Egger regression, and weighted-median estimator approach to assess the robustness of our results under relaxed model assumptions (34–36).

We conducted crucial sensitivity analyses to validate MR model assumptions. We excluded palindromic IVs that exhibited standard ambiguity, specifically those involving A/T or G/C SNPs with identical pairs of letters on both the forward and reverse DNA strands, as this ambiguity can affect strand identity. Additionally, we carried out a leave-one-out analysis, systematically removing one IV at a time and performing IVW with the remaining IVs to identify outlying instruments. To investigate the causal relationship between the genetic predisposition to psoriasis and obesity, as well as lipidemic traits, we conducted a bidirectional MR analysis. In this analysis, we utilized IVs for the outcomes to assess whether the “exposure” (which includes obesity and lipid-related factors, might have a causal effect on the “outcome” (in this case, psoriasis), as detailed in **Supplementary Tables 1–10**). The 18 psoriasis-associated independent loci with genome-wide significance were included as IVs in our reverse direction MR (**Supplementary Table 11**) and their effects were extracted from the respective obesity GWAS(s).

## Results

### Genetic correlation

After correcting for multiple testing (p<0.05/10), we found a significant positive genetic correlation between BMI and psoriasis (r_g_=0.22, p=2.44×10^-18^), and between WHR and psoriasis (r_g_=0.19, p=1.41×10^-12^). Given that BMI affects both traits in observation studies, we further investigated the genetic correlation between psoriasis and WHR_adj_BMI, in while the effect of BMI was controlled for. As anticipated, the positive genetic correlation was attenuated, reducing its original value (r_g_=0.07, p=1.81×10^-2^). This reduction in significance suggests that the shared genetic basis was primarily influenced by BMI but also exhibited a non-trivial extent independent of BMI. Regarding lipidemic traits, we did not observe any significant genetic correlation with psoriasis (apoA1: r_g_=-0.03, p=0.261; apoB: r_g_=-0.02, p=0.615; apoE: r_g_=0.09, p=0.721; HDL: r_g_-0.05, p=0.077; LDL: r_g_=-0.04, p=0.344; TC: r_g_=0.08, p=0.164; TG: r_g_=0.06, p=0.053). After excluding MHC region, the result was consistent with analysis from the entire region (**Table 1**).

**Table 1:** Genome-wide genetic correlation between obesity and lipidemic related traits and psoriasis.

Trait 1	Trait 2	r_g_	r_g__SE	p	r_g_MHCexcluded_	r_g__SE__MHCexcluded_	p__MHCexcluded_
BMI	psoriasis	0.22	0.03	2.44×10^-18^	0.23	0.03	2.44×10^-19^
WHR	psoriasis	0.19	0.03	1.41×10^-12^	0.19	0.03	2.15×10^-14^
WHR_adj_BMI	psoriasis	0.07	0.03	1.81×10^-2^	0.07	0.03	1.06×10^-2^
apoA1	psoriasis	-0.03	0.03	0.261	-0.04	0.03	0.170
apoB	psoriasis	-0.02	0.04	0.615	-0.02	0.04	0.639
apoE	psoriasis	0.09	0.26	0.721	0.07	0.26	0.779
HDL	psoriasis	-0.05	0.03	0.077	-0.05	0.03	0.040
LDL	psoriasis	-0.04	0.04	0.344	-0.04	0.04	0.343
TC	psoriasis	0.08	0.06	0.164	0.08	0.06	0.170
TG	psoriasis	0.06	0.03	0.053	0.06	0.03	0.039

rg, genetic correlation; SE, standard error; MHC, Major Histocompatibility Complex; BMI, body mass index; WHR; waist-to-hip ratio; WHR_adj_BMI, waist-to-hip ratio adjusted for body mass index; apoA1, apolipoprotein A1; apoB, apolipoprotein B; apoE, apolipoprotein E; HDL, high-density lipoprotein; LDL, low-density lipoprotein; TC, total cholesterol; TG, triglycerides.

### Cross-trait meta-analysis of obesity and lipidemic related traits and psoriasis

To identify individual SNPs influencing both obesity and lipidemic traits and psoriasis, we next conducted pairwise CPASSOC analysis. As summarized in **Table 2** (which is on page 10, line 394), we identified a total of 3 independent loci that were shared between BMI and psoriasis, 5 independent loci shared between WHR and psoriasis, 6 independent loci shared between WHR_adj_BMI and psoriasis, 7 independent loci shared between apoA1 and psoriasis, 10 independent loci shared between apoB and psoriasis, 8 independent loci shared between HDL and psoriasis, 5 independent loci shared between LDL and psoriasis, 8 independent loci shared between TG and psoriasis, and 5 independent loci shared between TC and psoriasis. However, no independent locus was identified between apoE and psoriasis. All these SNPs met the criteria of p_single trait_<1×10^-3^ and p_CPASSOC_<5×10^-8^. Notably, none of these SNPs identified by CPASSOC had been previously reported to be associated with psoriasis, nor with obesity or lipidemic related traits.

**Table 2:** Cross-trait meta-analysis between obesity, lipidemic-related traits and psoriasis.

SNP	CHR	BP	Alleles		BETA		p_trait_	p_psoriasis_	p_CPASSOC_	Genes within clumping area	MHC region
			A1	A2	trait	psoriasis					
BMI and psoriasis											
rs13062101	3	23483536	A	G	0.01	0.06	8.38E-06	3.82E-05	1.35E-08	UBE2E2	NO
rs6550679	3	18845347	T	G	0.01	0.06	6.21E-08	2.43E-04	4.41E-10	SATB1-AS1	NO
rs66733826	2	23582135	T	C	0.01	0.08	2.20E-06	3.53E-05	5.27E-09		NO
WHR and psoriasis											
rs2857106	6	32787570	T	C	-0.01	-0.09	1.60E-05	3.08E-07	4.40E-08	HLA-DOB, TAP2	YES
rs9263724	6	31104956	A	G	0.01	-0.08	8.78E-06	5.42E-08	5.19E-09	PSORS1C2, PSORS1C1, POLR2LP1	YES
rs9461366	6	27310533	A	G	-0.01	0.08	1.45E-06	9.09E-08	7.46E-09		YES
rs453779	6	32975381	A	G	-0.01	0.08	5.02E-05	1.36E-07	2.02E-08	HLA-DOA	YES
rs7761083	6	30434448	A	G	-0.01	0.08	2.57E-04	5.92E-08	1.03E-08	TMPOP1, SUCLA2P1	YES
WHR_adj_BMI and psoriasis											
rs10460566	2	25483121	A	G	-0.01	-0.08	2.61E-05	6.88E-05	3.56E-08	DNMT3A	NO
rs1894406	6	32787056	T	C	0.01	0.05	5.28E-06	2.86E-04	1.67E-08	HLA-DOB, TAP2	YES
rs2071543	6	32811629	T	G	0.01	0.09	2.13E-06	5.02E-05	1.14E-09	TAP1, PSMB8, PSMB8-AS1,	YES
rs453779	6	32975381	A	G	-0.01	0.08	4.03E-07	1.36E-07	6.86E-13	HLA-DOA	YES
rs56348466	11	63832984	A	G	0.01	0.08	1.05E-05	1.58E-04	2.09E-08	MACROD1, FLRT1	NO
rs6914721	6	34438564	A	G	0.01	0.07	6.49E-08	6.14E-05	5.27E-11	PACSIN1	YES
apoA1 and psoriasis											
rs12661831	6	28337769	A	G	-0.01	-0.13	5.00E-05	2.20E-06	2.10E-09	ZKSCAN3	YES
rs139786258	6	27243326	C	T	-0.01	0.19	1.40E-07	1.71E-07	4.02E-13		YES
rs143995422	6	28767690	T	C	0.01	-0.20	5.20E-08	3.40E-04	5.13E-10		YES
rs17189427	6	30750229	A	G	-0.01	0.13	7.30E-06	1.20E-04	1.36E-08	HCG20	YES
rs185758423	7	6538808	G	A	0.02	-0.15	2.80E-06	5.07E-04	2.63E-08	GRID2IP	NO
rs9295648	6	25186568	G	T	0.02	-0.06	3.90E-06	1.40E-04	8.88E-09	CMAHP	YES
rs9368794	6	34082335	G	A	0.01	-0.06	1.50E-06	2.33E-04	6.31E-09	GRM4	YES
apoB and psoriasis											
rs112067230	17	7303502	T	G	-0.01	-0.08	1.40E-05	4.24E-04	1.79E-08	TMEM256, NLGN2	NO
rs112647257	6	32804381	T	A	0.03	-0.14	3.90E-05	4.22E-05	4.95E-09	PSMB8, TAP2	YES
rs116039572	6	26231273	A	G	-0.01	0.20	2.90E-05	1.42E-06	1.25E-10	H1-3, H2AC9P, H3C6	YES
rs139561666	16	72761954	A	T	0.04	0.11	7.80E-06	4.52E-04	1.03E-08	ZFHX3-AS1, KRT18P18	NO
rs145162876	6	31104053	T	A	-0.01	-0.41	4.90E-05	4.83E-07	9.12E-11	PSORS1C2, PSORS1C1, POLR2LP1	YES
rs184114817	7	75653185	A	G	0.01	-0.24	1.90E-07	2.48E-04	1.00E-10	STYXL1	NO
rs190965527	5	131693015	T	C	-0.01	0.18	2.60E-05	5.21E-06	3.74E-10	MIR3936HG	NO
rs4148876	6	32796793	A	G	0.01	-0.16	5.10E-08	1.48E-07	5.20E-15	TAP2	YES
rs72801155	5	156016746	C	T	0.02	-0.20	1.20E-05	2.43E-04	8.27E-09	SGCD	NO
rs78426485	6	25342229	G	A	-0.02	0.18	2.50E-07	2.15E-04	1.10E-10	CARMIL1, CMAHP	YES
apoE and psoriasis											
NA											
HDL and psoriasis											
rs117965198	7	4662060	C	T	0.01	0.10	5.60E-06	5.71E-04	3.93E-08		NO
rs12661831	6	28337769	A	G	-0.01	-0.13	5.20E-05	2.20E-06	1.56E-09	ZKSCAN3	YES
rs139786258	6	27243326	C	T	-0.01	0.19	1.80E-06	1.71E-07	3.41E-12		YES
rs16871169	6	32910877	A	G	-0.04	0.44	3.70E-06	3.88E-04	1.75E-08	HLA-DMB	YES
rs184114817	7	75653185	A	G	-0.01	-0.24	9.30E-06	2.48E-04	2.58E-08	STYXL1	NO
rs453779	6	32975381	G	A	0.02	-0.08	2.60E-07	1.36E-07	3.23E-13	HLA-DOA	YES
rs61874865	10	122899616	A	C	0.03	-0.08	3.80E-06	8.51E-04	4.26E-08		NO
rs9295648	6	25186568	G	T	0.01	-0.06	2.60E-05	1.40E-04	3.92E-08		YES
LDL and psoriasis											
rs116039572	6	26231273	A	G	-0.02	0.20	3.00E-06	1.42E-06	2.06E-10	H1-3, H2AC9P, H3C6	YES
rs140159913	6	30885990	A	G	-0.01	-0.21	1.10E-05	2.09E-05	7.50E-09	GTF2H4, VARS2	YES
rs145162876	6	31104053	T	A	-0.01	-0.41	2.30E-04	4.83E-07	1.03E-08	PSORS1C2, PSORS1C1, POLR2LP1	YES
rs184114817	7	75653185	A	G	0.01	-0.24	6.00E-07	2.48E-04	5.07E-09	STYXL1	NO
rs28484982	4	3430770	C	G	0.01	-0.07	1.70E-07	1.95E-04	1.25E-09	RGS12	NO
TG and psoriasis											
rs114322499	6	29801180	T	C	0.01	0.11	1.20E-05	1.09E-06	1.86E-10	HLA-G	YES
rs117386389	8	71908473	A	G	-0.01	-0.75	6.30E-07	8.46E-04	6.45E-09		NO
rs139786258	6	27243326	C	T	-0.01	0.19	2.60E-05	1.71E-07	9.46E-11		YES
rs140430387	11	118402625	C	T	-0.01	-0.22	8.00E-07	5.66E-04	5.19E-09	TTC36, TTC36-AS1, TMEM25	NO
rs184114817	7	75653185	A	G	0.01	-0.24	1.00E-07	2.48E-04	2.93E-10	STYXL1	NO
rs190965527	5	131693015	T	C	0.01	0.18	1.80E-05	5.21E-06	1.19E-09	MIR3936HG	NO
rs75340846	6	30106437	G	A	0.01	0.20	3.30E-04	8.46E-07	8.29E-09	TRIM40	YES
rs77168598	11	55306145	T	C	0.01	0.10	4.40E-06	5.49E-05	2.43E-09	OR4C14P	NO
TC and psoriasis											
rs112813062	6	33771244	T	A	0.14	0.12	6.60E-04	9.18E-07	1.58E-08	MLN	YES
rs146461712	6	27913334	C	T	0.02	0.31	4.80E-04	3.63E-07	3.80E-09	ZKSCAN8, ZSCAN16-AS1	YES
rs3757186	6	28107662	T	C	0.03	0.18	1.60E-05	6.38E-07	1.49E-09		YES
rs4470843	6	23261366	C	A	0.04	-0.10	9.40E-05	3.98E-06	2.05E-08		NO
rs9379853	6	26357635	G	A	0.02	0.14	7.60E-07	8.81E-08	2.02E-11		YES

SNP, single nucleotide polymorphisms; CHR, chromosome; BP, physical position of SNP (base-pairs); A1, effect allele; A2, alternative allele; BMI, body mass index; WHR, waist-to-hip ratio; WHR_adj_BMI, waist-to-hip ratio adjusted for body mass index; apoA1, apolipoprotein A-1; apoB, apolipoprotein B; apoE, apolipoprotein E; HDL, high density lipoprotein; LDL, low density lipoprotein; TG, triglyceride; TC, total cholesterol; MHC, Major Histocompatibility Complex; NA, not available.

Due to the risk for horizontal pleiotropy, we analyzed the shared loci after excluding loci from MHC region. For BMI and psoriasis, among the 3 shared SNPs, rs13062101 was in proximity to UBE2E2, a gene linked to obesity and type 2 diabetes (37). For WHR_adj_BMI and psoriasis, the most significant shared SNP (rs56348466, p_CAPSSOC_=2.09×10^-8^) was near MACROD1 and FLRT1. The most significant shared SNP for HDL and psoriasis was also rs184114817 (p_CAPSSOC_=2.58×10^-8^), and the gene within clumping area was STYXL1. Similarly, as for the shared SNPs between apoB and psoriasis, the most significant one was also rs184114817 (p_CAPSSOC_=1.00×10^-10^). Among the 2 shared SNPs between LDL and psoriasis, rs28484982 was near RGS12, a gene that exhibited differential expression in visceral adipose tissue from morbidly obese patients (38). Among the 5 TG-psoriasis shared SNPs, rs140430387 was near TTC36, a gene identified as a key player in obesity (39). Notably, among the apoA1-proriasis and TC-psoriasis shared SNPs, none of them were found to be associated with dyslipidemia or obesity. Of note, some of the CPASSOC-identified significant SNPs were not mapped to any genes (8 out of 44). As a complementary method for cross-trait meta-analysis, the MTAG result between obesity and lipidemic related traits and psoriasis was shown in **Supplementary Table 12**. And a SNP list which combined the result from two cross-trait meta-analysis method was exhibited in **Supplementary Table 13**. Detailed annotations of each variant (after excluding MHC region) are provided in **Supplementary Tables 14, 15**.

### Pathway analysis

To explore the biological pathways represented by shared genes, we conducted an analysis to evaluate the enrichment of annotated genes identified by two cross-trait meta-analysis methods across obesity and lipidemic traits and psoriasis in KEGG, reactome pathway and Wikipathway analysis, and revealed several statistically significant shared pathways (p<0.05, **Supplementary Table 16**). Pathways for shared loci including progesterone-mediated oocyte maturation in KEGG analysis; mitotic spindle checkpoint, separation of sister chromatids, amplification of signal from the kinetochores, amplification of signal from unattached kinetochores via a MAD2 inhibitory signal, mitotic anaphase and mitotic metaphase and anaphase in reactome database; envelope proteins and their potential roles in EDMD physiopathology, arylamine metabolism, and brain-derived neurotrophic factor signaling pathway in Wikipathway analysis.

### Mendelian randomization analysis of obesity and lipidemic related traits and psoriasis

**Figure 2:**
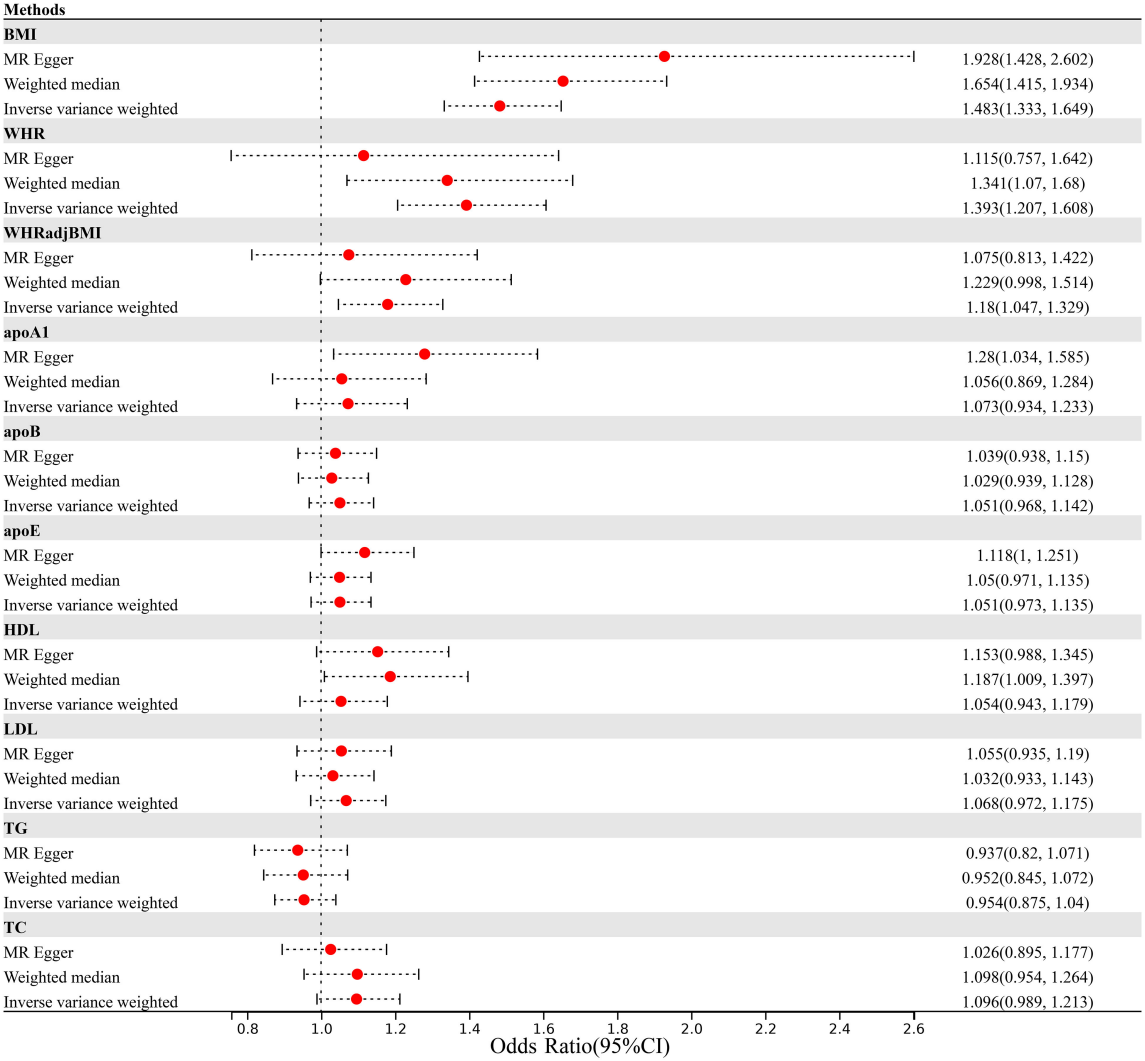
Mendelian randomization analysis estimating the causal effect of obesity and lipidemic traits on psoriasis after excluding MHC region. (MHC, Major Histocompatibility Complex; BMI, body mass index; WHR; waist-to-hip ratio; WHR_adj_BMI, waist-to-hip ratio adjusted for body mass index; apoA1, apolipoprotein A1; apoB, apolipoprotein B; apoE, apolipoprotein E; HDL, high-density lipoprotein; LDL, low-density lipoprotein; TC, total cholesterol; TG, triglycerides).

Finally, we performed a bidirectional two-sample Mendelian randomization analysis to investigate the potential causal relationship between obesity and lipidemic related traits and psoriasis. As illustrated in **Figure 2**; **Supplementary Table 17**, we found an elevated risk of psoriasis per-SD increment in BMI (IVW OR=1.483, 95%CI=1.333-1.649) using 510 IVs. The effect remained consistent when applying MR-Egger (OR=1.928, 95%CI=1.428-2.602) or Weighted median (OR=1.654, 95%CI=1.415-1.934) approach. We did not detect any evidence of horizontal pleiotropy (p for MR-Egger intercept=0.067).

Consistent with results from BMI, we identified a robust causal relationship with psoriasis using 328 WHR-associated IVs (IVW OR=1.393, 95%CI=1.207-1.608). However, although the MR-Egger analysis showed directionally consistent results, the significance was attenuate to null (MR-Egger OR=1.115, 95%CI=0.757-1.642). After accounting for the positive association of BMI, we still observed an increased risk of psoriasis with WHR_adj_BMI (IVW OR=1.18, 95%CI=1.047-1.329).

In contrast, we did not observe any significant association between lipidemic traits and the risk of psoriasis, as all effect sizes were close to 1.00 (apoA1, IVW OR=1.073, 95%CI=0.934-1.233; apoB, IVW OR=1.051, 95%CI=0.968-1.142; apoE, IVW OR=1.051, 95%CI=0.973-1.135; HDL, IVW OR=1.054, 95%CI=0.943-1.179; LDL, IVW OR=1.068, 95%CI=0.972-1.175; TC, IVW OR=1.096, 95%CI=0.989-1.213; TG, IVW OR=0.954, 95%CI=0.875-1.04).

Our findings were supported by the results of two critical sensitivity analyses. In addition, the leave-one-out analysis demonstrated that the observed causal associations between BMI and psoriasis, WHR and psoriasis, and WHR_adj_BMI and psoriasis were not influenced by outlier variants. As depicted in **Supplementary Table 18**, when systematically removing one IV at a time and conducting IVW analysis with the remaining IVs, the associations between BMI and psoriasis consistently centered around an OR of 1.46-1.50, while WHR and psoriasis associations centered around 1.38-1.42, and WHR_adj_BMI and psoriasis associations centered around 1.17-1.20. In contrast, associations between lipid-related traits and psoriasis consistently clustered around 1.00.

**Figure 3:**
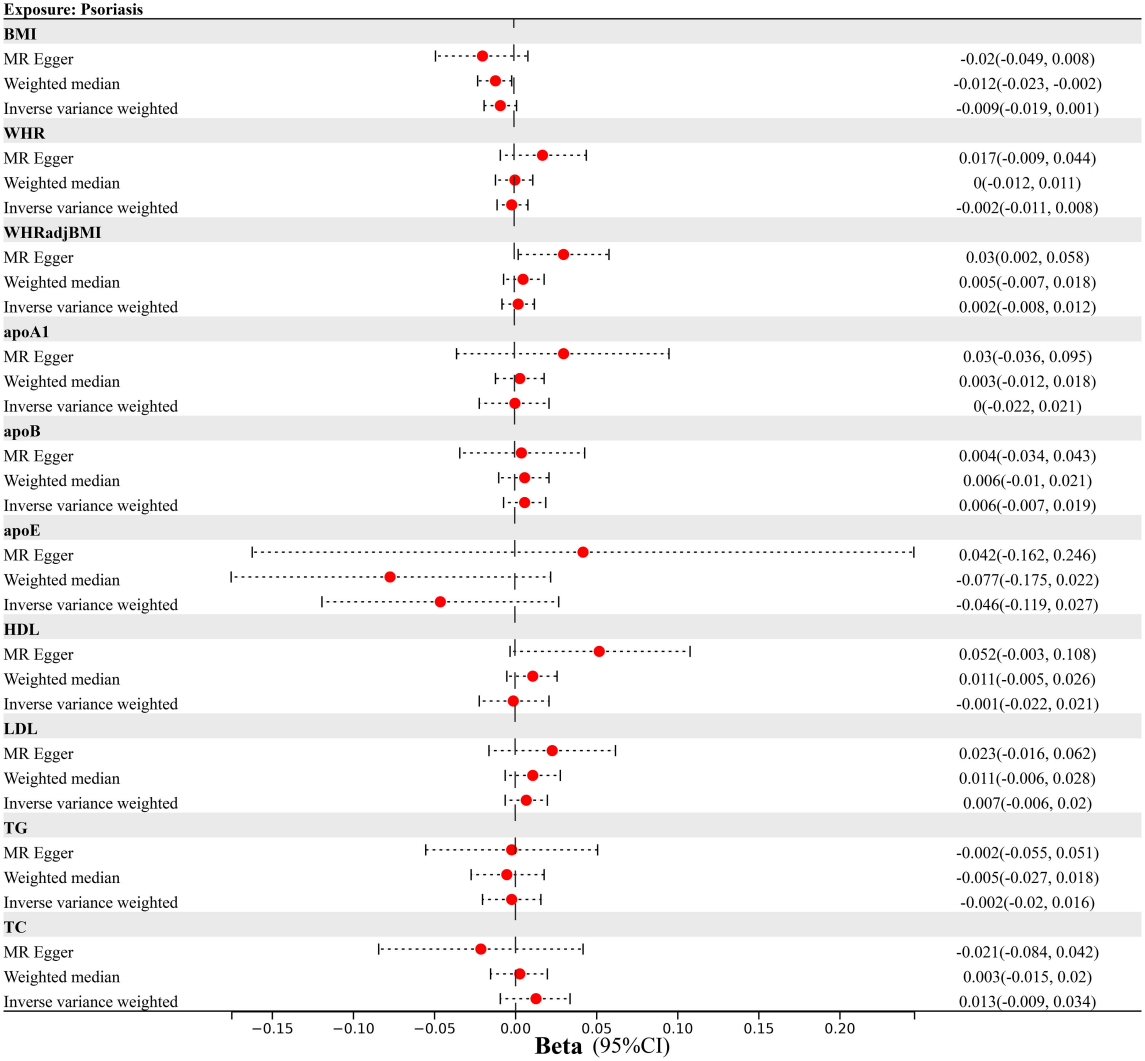
Mendelian randomization analysis estimating the causal effect of psoriasis on obesity and lipidemic traits after excluding MHC region. (MHC, Major Histocompatibility Complex; BMI, body mass index; WHR; waist-to-hip ratio; WHR_adj_BMI, waist-to-hip ratio adjusted for body mass index; apoA1, apolipoprotein A1; apoB, apolipoprotein B; apoE, apolipoprotein E; HDL, high-density lipoprotein; LDL, low-density lipoprotein; TC, total cholesterol; TG, triglycerides).

No impact of genetic predisposition to psoriasis on any of the obesity or lipidemic traits was observed in our reverse-directional MR analysis (BMI Beta=-0.009, 95%CI=-0.019-0.001; WHR Beta=-0.002, 95%CI=-0.011-0.008; WHR_adj_BMI Beta=0.002, 95%CI=-0.008-0.012; apoA1 Beta=0, 95%CI=-0.022-0.021; apoB Beta=0.006, 95%CI=-0.007-0.019; apoE Beta=-0.046, 95%CI=-0.119-0.027; HDL Beta=-0.001, 95%CI=-0.022-0.021; LDL Beta=0.007, 95%CI=-0.006-0.02; TG Beta=-0.002, 95%CI=-0.02-0.016; TC Beta=0.013, 95%CI=-0.009-0.034) (**Figure 3**; **Supplementary Table 17**).

## Discussion

This study performed a comprehensive genome-wide cross-trait analysis that thoroughly examines the shared genetic architecture and causal connections between obesity or lipidemic traits and psoriasis. Positive genetic correlations between BMI, WHR, WHR_adj_BMI and psoriasis were found. The utilization of CPASSOC revealed multiple shared loci among obesity, lipidemic traits, and psoriasis. Furthermore, MR analysis provided evidence of the causal role of obesity traits in the development of psoriasis.

The findings of this study are generally consistent with those of earlier conventional studies, yet provide novel insights in several important aspects. In addition to an overall improvement in statistical power, we applied a comprehensive analytical approach that was not available in prior studies. This included, for instance, the utilization of genetic correlation analysis to identify specific genomic regions contributing to both traits, as well as multiple functional analyses.

We applied advanced statistical methods to evaluate genetic correlations, utilizing publicly available summary statistics of genetic associations. Our analysis revealed positive genetic correlations between BMI, WHR, WHR_adj_BMI, and psoriasis, indicating a shared genetic basis for these traits. The connection between obesity-related traits such as BMI and an increased risk of psoriasis has been demonstrated through observational studies and MR analysis (15, 16, 21, 40, 41). Furthermore, the association between obesity and psoriasis has received affirmation from animal studies (42). A study that compared the genetic background of obesity in psoriasis patients with that of healthy controls through GWAS revealed a connection between 11 genetic polymorphisms and abnormal body weight in psoriasis patients (43). Findings from previous studies have also suggested that weight loss can lead to improved clinical outcomes, including a reduction in psoriatic flares (15, 44–46). Several potential mechanisms such as chronic inflammation, cytokine dysregulation, immune cell activation, and the influence of adipokines may account for this intricate association. For instance, adipose tissue in obesity generates and releases an increased amount of pro-inflammatory adipokines, including tumor necrosis factor- alpha (TNF-α), interleukin-6 (IL-6), and leptin, which results in skin inflammation and is associated with psoriasis (47, 48). TNF-α and IL-6 are related to the pathophysiology of psoriasis and serve as powerful therapeutic target (6, 49, 50). Leptin plays a pivotal role in bridging the pathogenesis of psoriasis and obesity by enhancing angiogenesis in psoriatic lesions, augmenting keratinocyte proliferation, and increasing the secretion of pro-inflammatory protein (48, 51, 52). Additionally, diminished serum adiponectin levels in psoriasis patients, along with a negative correlation with BMI, TNF-α, and IL-6 levels, suggest a potential anti-inflammatory role in inhibiting adipocyte adipogenesis and Th17 cell-mediated inflammation (53–56).

Cross-trait meta-analysis was conducted to further discover pleiotropic loci. In CPASSOC, we identified a total of 14 SNPs that were common between obesity traits and psoriasis, as well as 43 SNPs shared between lipidemic traits and psoriasis, indicating shared biological mechanisms underpinning the relationships among obesity, lipidemic traits, and psoriasis. Within these shared loci, we emphasize the significance of the locus of UBE2E2, RGS12 and TTC36 in relation to potential pathogenesis. UBE2E2, a gene associated with obesity and type 2 diabetes, encodes the ubiquitin-conjugating enzyme E2E2 expressed in adipose tissue, and it plays a regulatory role in insulin synthesis and secretion as well as adipocyte differentiation (37, 57–60). RGS12, which was differentially expressed in visceral adipose tissue from morbidly obese patients, and TTC36, which is involved in the occurrence and development of obesity, likely through the immune microenvironment, have both garnered recent attention (38, 39). In terms of lipid traits, we did not observe any significant genetic correlations with psoriasis. To the best of our knowledge, the most important shared genetic loci between lipidemic traits and psoriasis were not found to be strongly associated with lipidemic traits, and some of these loci were associated with obesity. Since dyslipidemia is one of the hallmarks of obesity, we can infer from these results that dyslipidemia might contribute to the risk of psoriasis by promoting the pathological processes that lead to obesity (52).

Pathways for shared loci included mitotic spindle checkpoint, separation of sister chromatids, amplification of signal from the kinetochores, amplification of signal from unattached kinetochores via a MAD2 inhibitory signal, mitotic anaphase and mitotic metaphase and anaphase in reactome database. These pathways were related to cell division. It’s crucial to note that psoriasis manifests as epidermal keratinocyte hyperplasia with proliferation, keratinocyte maturation and turnover rates (61). Cell division might be the potential mechanism underlying the causal relationships of obesity and lipidemic traits with psoriasis.

Genetic correlation provides valuable genetic insights into the observational associations by estimating the degree of pleiotropy or shared loci between two traits, while MR analysis helps establish causal relationships. Our findings suggest that obesity contributes to the pathogenesis of psoriasis, highlighting possible mechanistic relationships. Although strong observational relationship has been found between obesity traits and psoriasis, there has so far been little genetic or epigenetic overlap to support this. Recently, MR was performed to provide genetic evidence for causal link between psoriasis and other traits. Obesity traits have been identified to result in the development of psoriasis by the use of MR analysis, which is consistent with our findings (21, 62). Investigations into the association of lipidemic traits with the risk of incident psoriasis have yielded results differing from our findings (13, 22). The divergent findings observed in MR analysis can be ascribed to several potential factors. Psoriasis is a genetically intricate condition influenced by multiple genes, and MR analysis has the capacity to unveil specific gene associations or genetic variants that might not have received adequate consideration in previous investigations. Discrepancies in sample populations, encompassing demographic characteristics, environmental factors, and genetic profiles, can exert a substantial impact on MR analysis outcomes, possibly resulting in disparate results. Lastly, variations in study design or methodologies utilized in MR analysis may bolster sensitivity and precision, facilitating the identification of associations that were previously overlooked. Further research is warranted to validate the reproducibility and clinical significance of MR analysis results.

Several limitations need to be acknowledged. Firstly, as a complex disease, psoriasis is classified into four types, such as psoriasis vulgaris, pustular psoriasis, erythrodermic psoriasis and psoriatic arthritis. Additionally, it is categorized based on severity as mild, moderate, or severe. A larger sample size specific to psoriasis subtypes, gender, age of onset, or related phenotypes are required to identify more novel shared loci. Moreover, the generalizability of our findings regarding the shared genetic basis is limited to the European population. Further genome-wide association studies involving diverse ethnic groups are warranted to enhance our understanding of the genetic factors at play. Secondly, while the statistical power of our study and the new analysis method has significantly improved compared to previous analyses on the same topic, it is important to acknowledge that the phenotypic variance explained by IVs for some traits remains relatively modest. Therefore, future studies with even greater statistical power are warranted. Nevertheless, it’s worth noting that our instruments demonstrated sufficient strength, as reflected by F-statistics. Finally, the findings identified by our study relied exclusively on functional datasets and algorithms. In-depth experimental investigations are necessary to gain a deeper understanding of the underlying pathophysiological mechanisms.

In conclusion, the present study utilized GWAS summary statistics and advanced statistical genetics approaches to expand upon previous observational associations. An intrinsic link between obesity-related traits and psoriasis has been demonstrated. No significant genetic correlation and causal relationship was found between lipidemic traits (HDL, LDL, TG, TC, apoA1, apoB, apoE) and psoriasis. The genetic correlation and causal role of obesity-related traits (BMI, WHR, WHR_adj_BMI) in psoriasis highlight the significance of weight management in both the prevention and treatment of this condition. Further research is essential to comprehensively characterize the heritable component of obesity and lipidemic traits in the context of psoriasis.

## Data availability statement

The datasets presented in this study can be found in online repositories. The names of the repository/repositories and accession number(s) can be found in the article/**Supplementary Material**.

## Author contributions

YW: Conceptualization, Data curation, Formal analysis, Methodology, Writing - original draft. MH: Conceptualization, Data curation, Formal analysis, Methodology, Writing - original draft. XC: Data curation, Investigation, Writing - review & editing. JW: Data curation, Writing - review & editing. LL: Data curation, Writing - review & editing. JW: Data curation, Supervision, Writing - review & editing. CL: Funding acquisition, Supervision, Writing - review & editing. LH: Funding acquisition, Supervision, Writing - review & editing. YL: Funding acquisition, Supervision, Writing - review & editing.
